# Scattering of spoof surface plasmon polaritons in defect-rich THz waveguides

**DOI:** 10.1038/s41598-019-42412-6

**Published:** 2019-04-18

**Authors:** Andreas K. Klein, Alastair Basden, Jonathan Hammler, Luke Tyas, Michael Cooke, Claudio Balocco, Dagou Zeze, John M. Girkin, Andrew Gallant

**Affiliations:** 1 Department of Engineering, Durham University, South Road, DH1 3LE Durham, UK; 2 Department of Physics, Durham University, South Road, DH1 3LE Durham, UK

**Keywords:** Metamaterials, Terahertz optics, Nanophotonics and plasmonics

## Abstract

We report on the first observation of ‘Spoof’ Surface Plasmon Polariton (SPP) scattering from surface defects on metal-coated 3D printed, corrugated THz waveguiding surfaces. Surface defects, a result of the printing process, are shown to assist the direct coupling of the incident free-space radiation into a spoof SPP wave; removing the need to bridge the photon momentum gap using knife-edge or prism coupling. The free space characteristics, propagation losses and confinement of the spoof SPPs to the surface are measured, and the results are compared to finite-difference time domain simulations. Angular resolved THz spectroscopy measurements reveal the scattering patterns from surfaces and are compared with Mie theory, taking into account the shortened wavelength of the photons in their bound SPP state compared to their free space wavelength. These results confirm yet another similarity between the properties of THz spoof SPPs and their natural, non-spoof, counterparts at optical and infrared frequencies which also, unexpectedly, adds functionality to the structures.

## Introduction

The field of plasmonics offers the possibility for sub-wavelength manipulation of electromagnetic radiation which can lead to more compact and efficient optical devices or optical circuits^1–3^. The concept of spoof-SPPs, where a surface structured on a sub-wavelength scale supports modes with cut-off frequencies determined by the geometry, allows the mimicking of SPPs and extended plasmonics to virtually all frequencies^4,5^. While the study of spoof SPPs is a very active field of research, most of the devices demonstrated to date work at microwave frequencies as their dimensions are suitable for low-cost printed circuit board type fabrication^6–10^. Spoof-plasmonic structures are also appealing for many photonic applications, with advanced properties such as switching^11^ and beamforming reported^12^. Given that bound quasi-particles, such as an SPP, have a non-linear dispersion relation that lies to the right of the light line, additional momentum is required for a photon to couple from free space to the surface to form the SPP. This momentum gap is usually bridged with either prism coupling or scattering. However, the structures presented here do not require any additional means to couple radiation from free space, which we suggest is due to the high defect density on the surface offering a multitude of scattering opportunities. This effect is well known for classic, optical SPPs^13^, but has not been reported for spoof SPP structures. This is reasonable because, for operation in the microwave region, spoof SPP structures which possess feature sizes of the order of centimeters, are unlikely to include defects on the same scale. At higher (THz) frequencies, where wavelengths are of the order of tens of microns, semiconductor fabrication techniques are commonly used to produce the spoof SPP structures and are thus also usually free from defects comparable in scale to the used wavelengths. But 3D printing, which has been reported for millimeter wave^14^ but not for spoof plasmonic THz structures before, does exhibit a high defect density as the feature sizes are pushing the limits of the fabrication technique. 3D printing has high commercial potential where it competes with other serial techniques, such as milling, and can produce low cost and lightweight objects. One example is in the production of waveguides for high-frequency applications. Metalized, low-density 3D printed parts can replace commonly used materials such as brass. For example, 3D printed, metal-pipe rectangular waveguides capable of operating up to 1.1 THz have recently been reported^15^. In the following we present a detailed characterization of the 3D printed THz spoof plasmonic waveguide which demonstrates the scattering of the bound spoof plasmons at the defects and an explanation with the classic Mie theory and FDTD simulations.

## Results

Four 3D printed spoof SPP structures with different geometric properties and defect densities are investigated. The geometrical and plasmonic properties, i.e. the propagation length and confinement, are summarized in Table 1 and compared with other spoof plasmonic structures from literature. The geometry of the samples investigated here are discussed in detail in the Supplementary Materials, including the relationship between the different geometric parameters and the transmission curve obtained. For the later discussion of the performance of the samples, literature values of different THz SPP structures are included as well. The structures were first characterized to obtain their plasmonic properties, i.e. the transmission spectrum, which shows the geometry dependent cut-off, and knife-edge scattering experiments to determinate the propagation length and confinement of the spoof plasmons to the surface. This was followed by the determination of the defect density with scanning electron microscopy and an optical surface profilometer. Finally, the scattering of the THz spoof plasmons at the defects was measured with angular resolved THz spectroscopy.

**Table 1:** Geometric parameters and characteristic lengths of the 3D printed SPP structures in comparison with literature values.

Sample	Geometrical Parameters [µm]			Propagation Length *L*_*x*_		Confinement *L*_*z*_		Defect PSD signal ratio	Unassisted free space coupling
	p	d	h	µm	λ	µm	λ		
1	150	50	30	706 ± 181	~2.4	75 ± 4	0.25	2.1	Yes
2	150	50	35	384 ± 23	~1.3	119 ± 5	0.4	6.8	Yes
3	165	50	30	478 ± 32	~1.5	75 ± 4	0.23	7.3	Yes
4	75	30	15	590 ± 36	~2	69 ± 2	0.23	NA	Yes
ref.^26^	Slots in metal film @ 0.28 THZ			~80000	~75	~1690	~1.6		No
ref.^27^	Metamaterial array @ ~1 THz			~50000	~176	~600	~2		No

It is apparent that while the SPPs are highly confined, the propagation length of the 3D printed structures is strongly reduced compared to the literature values from structures with good surface quality. p: pitch, d: grove thickness, h: height as indicated in Supplementary Materials. Defect Power Spectral Density (PSD) signal ratio: Ratio between defect peak and corrugation peak.

### THz characterization

The non-linear dispersion relation of SPP results in an increased momentum of the quasi-particle, in comparison to a free space photon, near the cut-off frequency. There are two common ways to bridge the momentum gap: high refractive index prisms, and scattering^13^. The latter technique, called knife-edge scattering in experimental setups, is favorable for broadband SPP structures because a wider range of frequencies can be addressed simultaneously as the scattering offers a wide range of momenta. The 3D printed structures investigated here, however, do not require any such coupling techniques in contrast to previously reported THz spoof SPP structures. We assume that this property arises from the defect-rich surface. The Fourier decomposition of a rough surface contains many different wave vector components, enabling the excitation of many different SPPs over a wide range of angles simultaneously. The wide range of momenta available directly after the scattering at wavelength-sized defects results in the potential launch of SPPs at every defect. The coupling efficiency can be qualitatively evaluated by the attenuation at the cut-off frequency which lies in the order of 20–30 dB, which is comparable to values of knife-edge scattering on defect-free samples fabricated with photolithography. Although surface roughness as a means to launch SPPs is well known for optical frequencies, it has not previously been reported for spoof SPPs^16–19^.

**Figure 1:**
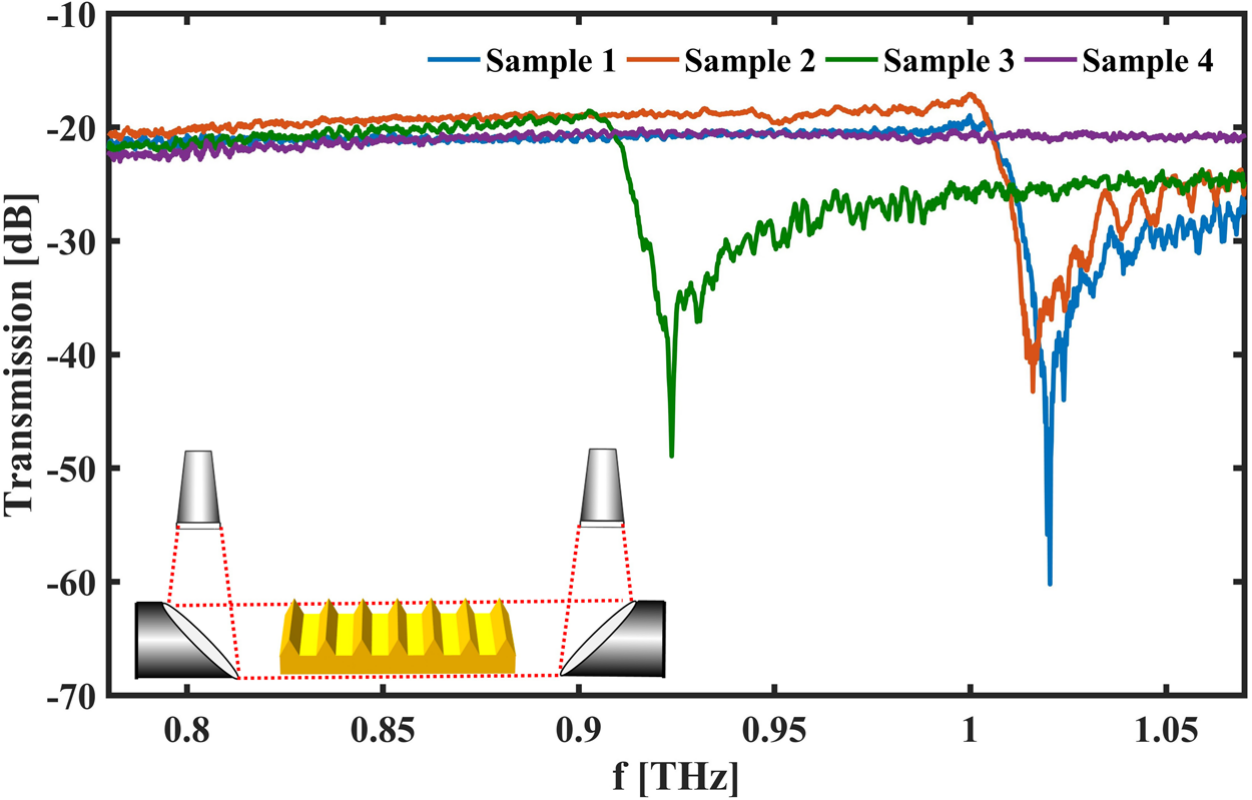
Transmission spectra of 3D printed spoof SPP structures with a triangular cross-section measured without additional means of coupling. A schematic of the measurement setup is shown in the inset. The THz beam originating at the horn antennas is collimated by a parabolic mirror, propagates across the surfaces and is then focused at the receiving horn antenna by a second parabolic mirror. The spectra agree very well with the results of the FDTD simulations (see Supplementary Material). FDTD simulation of sample 4 shown in has simulated a cut-off frequency around 1.5 THz and, therefore the sample exhibits a flat spectrum in the measured range.

The transmission spectra in Fig. 1 were recorded with the samples placed in the collimated part of the THz beam with a diameter of 10 mm. The key characteristics of SPPs are the propagation length and the confinement of the SPP to the surface^20^. Both characteristic lengths can be obtained by variations of the knife-edge scattering arrangement (see methods and Supplementary Materials) and the results can be found in Table 1.

### Defect density

**Figure 2:**
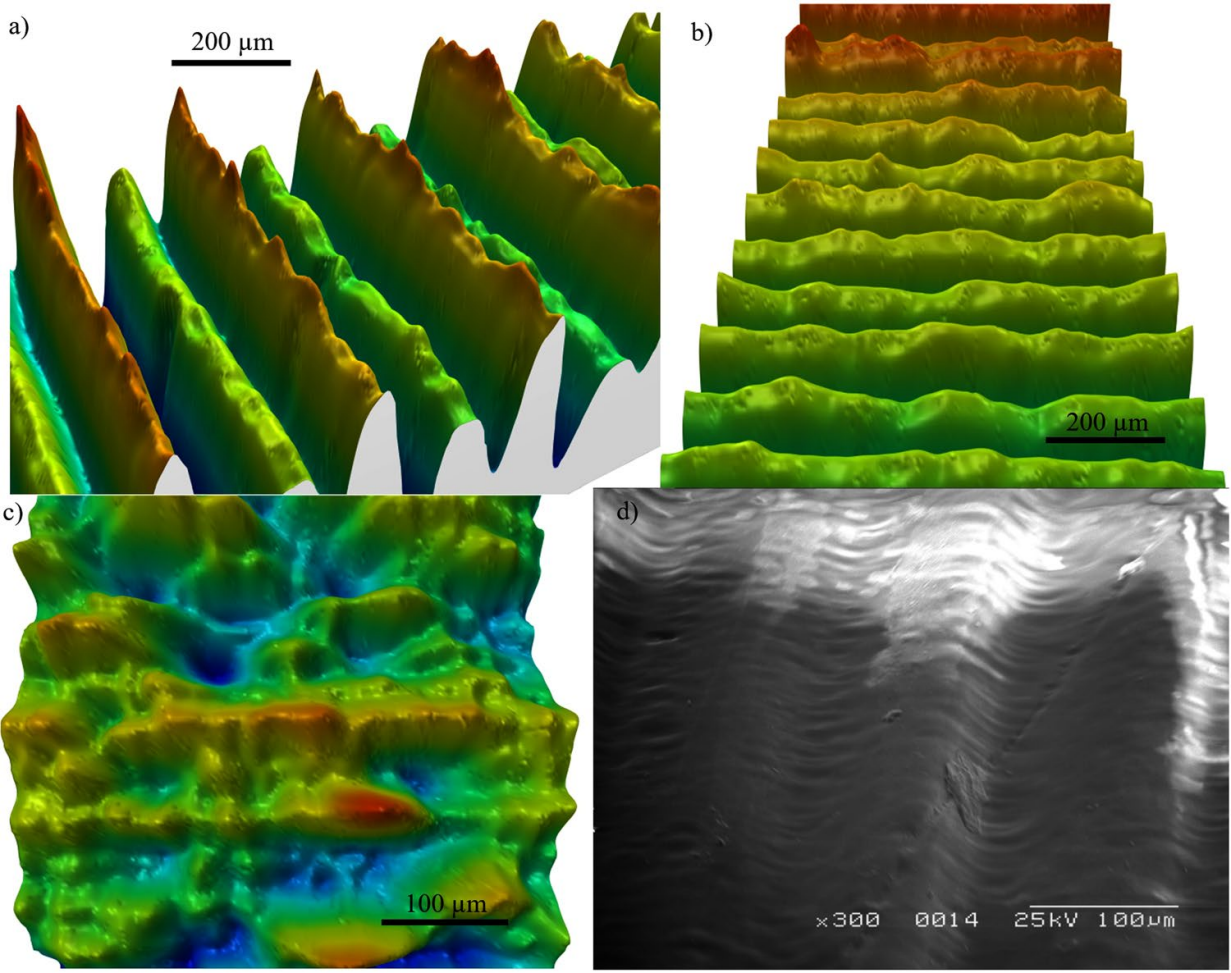
(**a**–**c**) 3D maps from the optical profilometer measurements. (**a**) shows the alternating height on sample 3. (**b**) shows the surface roughness on the top of the ridges. (**c**) while the presence of grooves on sample 4 is visible, the defects are in the same size order as the periodicity. (**d**) The micrograph of sample 4 on the other hand, gives the impression that there is a periodicity that could support the measured spoof THz SPPs.

The high defect density of the 3D printed samples is apparent when looking at the micrographs in Fig. 2, where various types of defects visible. For example, there are deformations to the surface along the ridges, probably originating from the stepwise printing process; defects where the grooves are damaged or incomplete; and debris, most likely originating from excess resin that forms irregularly shaped objects, on the surface. The surface profiles allow the average power spectral density (PSD) to be calculated which, in turn, gives access to the statistics of the surface roughness. The detailed results of the PSD and SEM micrographs can be found in the Supplementary Materials.

The PSD spectra can be used to quantify both the quality of the periodic SPP structure and the defect density. The periodicities of sample 1–3 are in good agreement with the target parameters listed in Table 1, with 150 µm for sample 1 and 2 and 166 µm for sample 3 but structure 4 shows no periodicity. The Full Width Half Maximum values for the first three samples are 8, 7.4 and 9.4 µm respectively, meaning that the periodicity has a deviation of ±3%. Furthermore, the PSDs of all structures have an above zero amplitude at most other wavelengths, indicating a broad range of defects. Besides the prominent peak at the wavelength of the periodicity, there is a very well defined feature at shorter wavelengths and some small peaks scattered over the spectrum. The 3D maps show that this part of the PSD is indeed caused by the defects on the surface. The maximum of the defect peaks is between 15 and 20 µm and they all have a FWHM of ~8 µm. While the PSD can provide information on a surface, e.g. the surface roughness, it is not possible to extract a defect density. It is, however, possible to obtain a quantity that gives a good impression on the number of defects on the surface by comparing the ratio between the contribution of the defect signal and the corrugation signal of the PSD. This ratio varies from 2.1 for sample 1, which therefore is the defect poorest, up to 7.3 for sample 3, which is the defect richest. Sample 4 could not be evaluated this way; the PSD as a method of evaluation struggles with the smaller sizes and becomes meaningless when the surface roughness is of the same length scale as the feature height. Even the autocorrelation function in the PSD process cannot discriminate between the periodic signal and the noise when the defects are dominant.

### Angular resolved THz measurements

**Figure 3:**
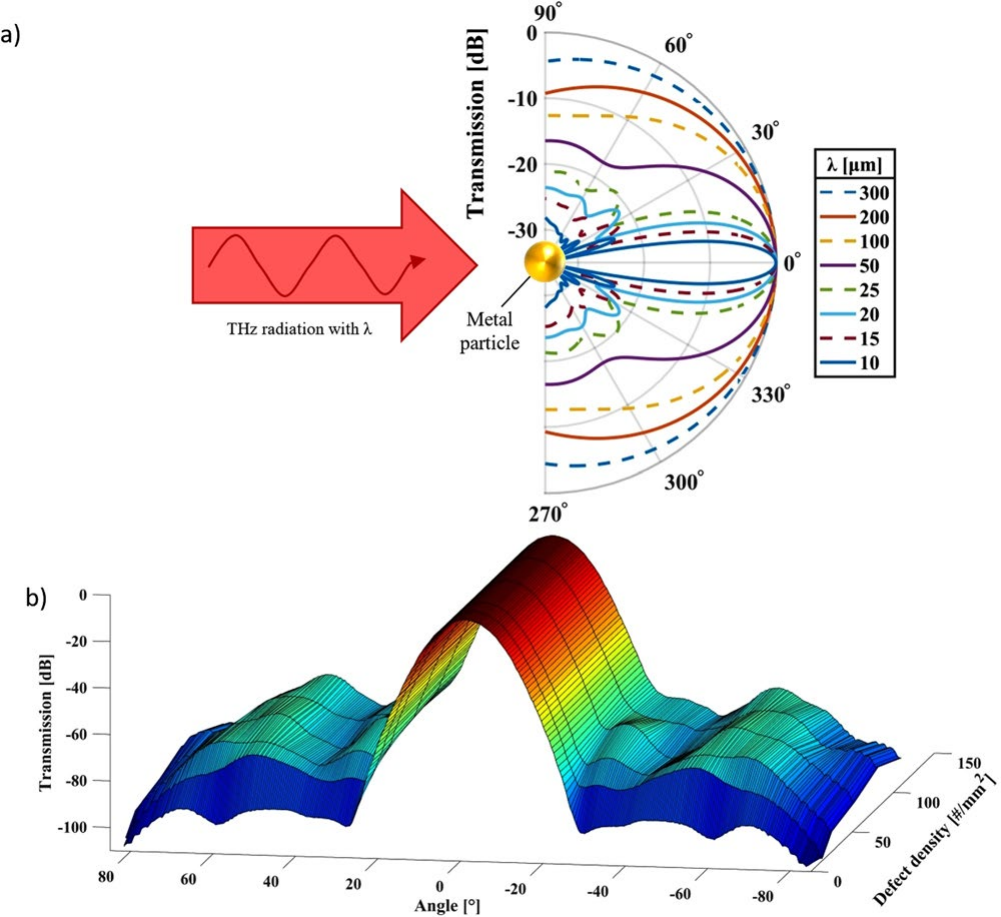
(**a**) The forward scattering of THz radiation with different wavelengths incident on a metallic sphere with a radius of 20 µm as predicted by the Mie theory (from FDTD simulation). (**b**) Simulation of the angular scattering pattern of a defect rich sample with different defect densities.

The scattering at defects on surfaces or rough surfaces are well studied for nanometer scaled defects and roughness at optical frequencies^21^. The properties of defect rich spoof plasmonic surfaces, however, have not been previously studied, as many spoof plasmonic experiments are conducted at microwave frequencies where no unintended defects due to manufacturing tolerances occur and defects are usually well defined and intended^22,23^. As a first approximation, the defects can be regarded as metallic spheres and as the THz radiation propagates in the plane of the sample surface only, the well-known case of Mie scattering on a metal cylinder applies^24^. We focused on the forward scattering properties, as these are the ones that we can determine experimentally, due to the geometrical constrains caused by the size of the THz transceivers. Figure 3 shows the Mie scattering at perfect electric conducting sphere with a radius r = 20 µm for different wavelengths (from FDTD simulations). When the sphere is significantly smaller than the wavelengths (r < 1/5λ) the scattering becomes less omnidirectional than at longer wavelengths where there are no striking features in the curve. For wavelengths of the order of the radius, forward scattering dominates, but with pronounced side lobes. These side lobes are known to increase in intensity and scatter more towards the forwards direction when the particle is placed on a substrate^25^. If we apply these insights to the present situation of scattering from defects on spoof plasmonic surfaces at THz frequencies, it can be expected that a first scattering event of a free-space photon will result in a nearly omnidirectional scattering, as the free space wavelength is much larger than the measured defects. The spoof plasmonic surface, however, has a preferred direction of propagation across its corrugation where it is able to support SPPs. Therefore, the omnidirectional scattering only excites an SPP in the direction across the corrugation. The SPP waves have smaller wavelengths than their corresponding frequency in free-space and, therefore will exhibit a different scattering pattern than the free-space wave for the same defects. As the samples have a high defect density, the effect of multiple scattering events needs to be taken into account along with the polydispersity of the defects. An increasing defect density leads to a reduction in the lobe pattern which is replaced by an increasingly uniform field pattern^21^. Furthermore, the polydispersity also decreases the visibility of the lobed pattern, as similar but not identically sized scatterers will have coinciding minima and maxima, as seen in Fig. 3a for wavelengths 10–25 µm. The results from FDTD simulations of a defect-rich SPP waveguide in Fig. 3b show that while the scattering intensity strongly increases with number of defects from a sparse to a defect rich surface, the scattering intensity saturates quickly and the weak lobe pattern that is visible for lower defect densities disappears into wide, uniform lobes. Additionally, the scattering lobes move towards the center with increased defect density. All this is in agreement with the previously described behavior at optical frequencies of multiple scattering^21^.

**Figure 4:**
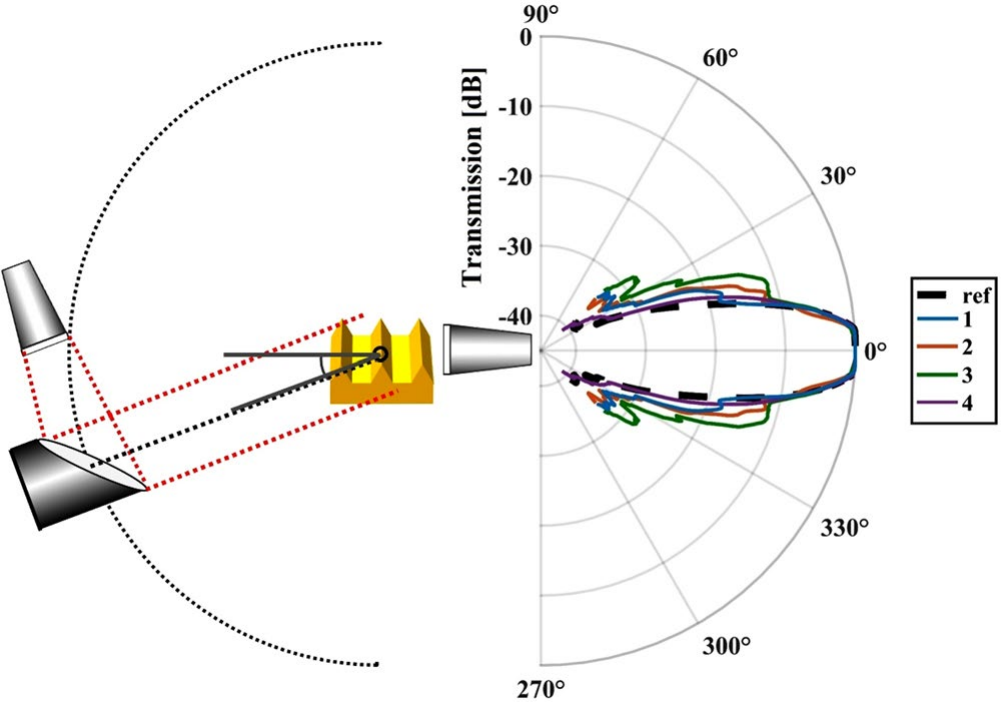
Schematic of the angular measurement setup (left) and the polar plot of the results (right). The angular plot shows the averaged intensity below the cut-off frequency of the individual samples. All SPP samples show an increased acceptance angle. Additionally, side lobes as they are known from Mie scattering are visible.

Angular measurements were obtained over a range of ±45°; a schematic of the experimental setup is shown in Fig. 4. The polar plot in Fig. 4 shows that all four samples have an increased acceptance angle compared to the bare horn antenna used as a reference. Additionally, multiple side lobes are visible, which resemble the shape of the previously shown Mie scattering plots. The SPP interaction area is several mm^2^, which will typically include 100 s of defects. Therefore, the individual defect positions and sizes become less relevant and, instead, the defects can be considered as a collective. This statistical nature of the scattering leads to the side lobe symmetry. The side lobes are most pronounced for samples with the highest defect concentration, with sample 2 and 3 showing the first side lobes just below the −10 dB mark, which is in good agreement with the simulations that quickly saturate in their side lobe intensity, which explains why the intensities are very similar despite having different defect densities. Additionally, the side lobes of sample 1 and 3 are nearly identical in shape and only vary in intensity, which again agrees with simulations where the side lobe shape is preserved for lower defect concentrations. While sample 3 has an even higher defect concentration which is expected to result into a broadening of the side lobes, the broadening could be also attributed to the larger defects found in the PSD for this sample, especially since some additional side lobes at higher angles are visible as well.

**Figure 5:**
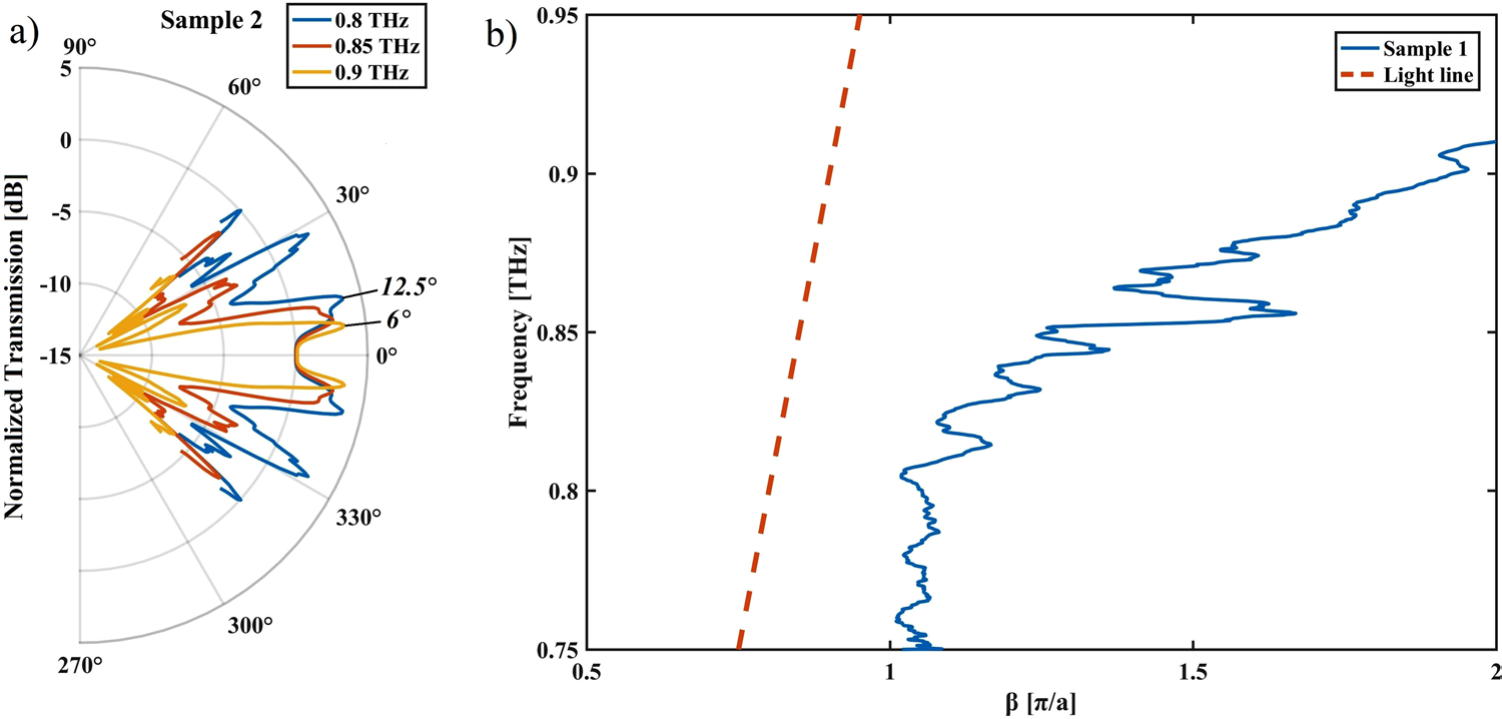
(**a**) Frequency resolved angular plots show that the scattering lobes move toward the forward direction with increasing frequency. Due to normalization with the bare horn antenna, the forward scattering lobe is removed. (**b**) The dispersion curve shows that the bound wavelength of the sample.

The frequency resolved plots of the angular resolved measurements in Fig. 5a reveal Mie like scattering characteristics similar to the theoretical case in Fig. 3a: When varying the frequency within the narrow band below the cut-off frequency of the SPP waveguide, we see that the scattering side lobes change their angle increasingly towards the forward direction. Despite the narrow frequency range, the scattering angles of the forward scattering lobes changes from ~6° for the highest frequency (0.9 THz) to ~12.5° for the lowest frequency (0.8 THz), while according to the Mie theory, there would be hardly any change expected for a free wave scattering from particles of this size.

This is an indicator that the wavelength of the scattering wave has to be significantly shorter than the free space wavelength, as otherwise the scattering pattern would be omnidirectional and would hardly vary with such small wavelength changes of less than 10% of the wavelength.

It is challenging to directly measure the bound SPP wavelength as it is impossible to directly measure the wave vector *k* ≡ 2*π*/λ. It is, however, possible to extract the phase constant *β* from S-parameters, which is proportional to |*k|* where *β* is the real part. As expected, the dispersion curve in Fig. 5b), shows an increasing *β* as the frequency approaches the cut-off frequency. Due the narrow frequency band (0.75 to 1.1 THz) of the THz vector network analyzer we cannot observe the convergence of the SPPs dispersion curve and the light line at lower frequencies. However, the dispersion curve does indicate that the SPP wavelength is around half the length of the wavelength of a freely propagating wave at the same frequency.

## Discussion

The most general approach to interpret the results of the angular measurements is to compare them to Mie theory. Whilst this comparison has to be of a qualitative nature, as Mie theory usually accounts for spherical (or cylindrical) uniform particles, which is not the experimental reality as the micrographs show, the shape of the measured polar plots still closely resemble Mie scattering. The similarities are even larger when taking multiple scattering events into account. The observation of the side lobes at these defect sizes is interesting in itself, as the majority defects are in the order of 5–10% of the free space wavelength and one would usually expect a uniform scattering pattern without lobes and with equal contributions to the backward and forward scattering. However, the observed scattering pattern shows more similarities to the scattering typically found with wavelength-sized defects. The most likely explanation is that we observe the scattering of the bound SPPs, which have a much shorter wavelength than their free space equivalent, but the THz radiation still has to couple to the surface first. The observed pattern, therefore, would be a convolution of the scattering of the more omnidirectional scattering of the free-space wave on particles much smaller than the wavelength and the scattering of the bound SPP wave on the same defects, but this time the wavelength is in the same order as the defect size. This fits with the observed changes in the different defect densities and distributions. It is worth noting that the two samples with similar defect size distributions (1 and 2) have the same shape of side lobes, and the two samples with the same defect concentration have the same amount of scattering/similar intensity of their side lobes (2 and 3), but different shapes of the scattering pattern.

Table 1 shows that while the SPPs are strongly confined to the surface of the 3D printed structures, the propagation lengths are only of the order of the wavelength itself. The confinement is between 69 µm and 119 µm, which is a third to a quarter of the free-space wavelength and in good agreement with the FDTD simulations which show an extension of ~70–110 µm. This confinement is approximately four times higher than that for previously reported THz SPP structures^26,27^, but the propagation length is just greater than two free space wavelengths, which is extremely short in comparison to the order of tens^26^ or hundreds^27^ times the wavelength in literature. The sample with the lowest defect concentration has the longest propagation length, indicating that the short propagation length is caused by the defects. Whilst enabling unassisted free space coupling, the defects also cause constant decoupling of the SPPs, resulting in high radiative losses, and hence very short propagation lengths. Even if the unassisted coupling is very useful for interconnect applications, the attenuation is of the order of 10 dB/mm and which is too high for waveguides. A better control of the printing quality, or potentially some post processing, would circumvent this issue by having a defect free waveguide with only small sections with defects to couple in and out. A better control of the print quality also opens up the possibility of tailoring the receiver characteristics, e.g., by making forward scattering dominant or tailoring the acceptance angle of the structures to minimize cross-talk in highly integrated photonic circuits.

## Conclusion

We have demonstrated the free space coupling of THz radiation to 3D printed defect-rich spoof SPP surfaces. While the SPPs are well confined to the surface, the propagation lengths are only in the order of a few free-space wavelengths. The scattering patterns of the structures have been investigated by comparing them to Mie theory, which revealed that the scattering is dominated by the bound SPPs rather than the free space waves. To the best of our knowledge, this is the first study on scattering of spoof SPPs.

The resulting observed unassisted free space coupling of radiation has great potential for highly integrated photonic circuits, as no other components to couple are necessary and the radiation patterns can be tailored to the applications needs, e.g. to minimizing cross-talk or for frequency selectivity. For this, improvements in the printing quality are necessary for a better control over the surface quality and intended “doping” with specific defect concertation and distributions.

## Methods

The samples were produced with a Formlabs Form 2 stereolithography 3D printer using Clear Resin v2 (GPCL02) with a nominal resolution of better than 25 µm and subsequently coated with approximately 300 nm of gold to have a metal layer thickness above the skin depth which is less than 100 nm at 1 THz. The roughness measurements were undertaken with a Taylor Hobson CLI-2000. As the term PSD is loosely defined and is displayed with different units on the x- and y-axes^28^, we will briefly elaborate on the specific technique employed here. We use an averaged PSD, which has wavelength rather than the frequency on the x-axis, and is particularly useful for surface metrology applications as the wavelength correlates to the feature size. To evaluate the quality of the periodicity, we have used an anisotropic PSD which only includes horizontal spectral components, i.e. cutting across the corrugations along the direction of propagation. To identify the relationship between quality of the target feature (i.e. the period of the grooves) and all other features on the surface, an isotropic PSD is used which includes spectral components from all directions. The parameters typically extracted from the PSD, such as surface roughness, are of limited use for a corrugated film, as the periodicity itself is the main contributor to it, and the defects would only be a deviation of the ideal value. However, the histograms of the PSD do show the distribution of features by their size and the intensity. A PSD of an ideal structure, for example, would only exhibit one infinitely sharp peak at the frequency of the chosen periodicity. A broadened peak at the wavelength of the periodicity indicates deviation from the periodicity, amplitude at any other wavelengths are indicative of defects. The PSD spectra can be found in the Supplementary Materials.

For the FDTD simulation (Software: Lumerical FDTD), a workaround was used to address the issues accompanied by the attempt of simulating a realistic defect-rich SPP sample. It is challenging to generate a realistic model as it requires a significant level of computational resource for multiple reasons: To simulate Mie scattering a single defect has to be mapped with multiple mash points; the simulated domain has to have a certain minimum size to allow multiple scattering; and the area has to be large enough to be of statistical significance, so that the positioning of individual defects does not significantly change to results. A simulation fulfilling all these requirements would require terabytes of RAM. To realize a simulation on the available workstation with 128 GB RAM a smaller area of the SPP waveguide with randomly distributed defects is simulated. This step is repeated multiple times with different random distributions of the defects until the results converge and do not show significant change with the next iteration. There are some considerable differences between the FDTD simulations and the experimental measurements: In the FDTD simulation a wave is launched at one end of the structure and then a far field projection of the electric fields at the other end of the structure results in the plots in Fig. 3; in the experiment, the source rotates around the sample and the receiver is stationary, i.e. the THz wave propagates in the opposite direction. Whilst Maxwell’s equations are time reversible, the order in which the scattering events take place might matter. Unfortunately, neither an angular resolved FDTD simulation nor swapping receiver and transmitter in the measurements is feasible because of the vast computational resources required or the increased loss of signal respectively. All THz characterization has been conducted with a THz vector network analyzer (VNA) comprising a microwave VNA with a frequency range up to 42.5 GHz and two Virginia Diodes extenders (VNAX WR1.0) with a frequency range from 0.75–1.1 THz. The transmission spectra are measured by placing the 3D printed devices in the collimated part of the beam between two parabolic mirrors without any additional means to couple from free space. The same measurements were repeated with the antennas pointing at an angle of 60° to the surface normal, to exclude the possibility that the free-space coupling happens due to edge-firing^13,20^. Knife-edge scattering was used in the characterization to have defined points to couple in and out. These knife-edge scattering experiments were carried out by placing the transmitting and receiving horn antennas at a 60° angle to the sample surface normal, aiming at the gap between the sample and in-coupling (or respectively outcoupling) knife from a distance of less than 5 cm. A schematic of the knives’ positions relative to the sample is shown in Fig. 4. For the propagation length measurements, one knife is placed near the sample and kept in a fixed position to couple the radiation in while a second knife is placed parallel to the first one to couple out. The distance between the two knives is incrementally increased, and the spectra for each increment are recorded. A third knife was placed between the two knives to reduce transmission through the direct line of sight to ensure that only the contribution of the SPPs is measured. The configuration for confinement measurements was similar but the outer two knives used for coupling in and out are kept at a fixed position, and only the middle knife was moved perpendicular to the surface. Illustrations on the measurement principle and the results of the fittings can be found in the Supplementary Materials.

For the angular measurement, the sample was placed at the pivot point, and the collimated beam is rotated around it in a fixed distance, as the receiving antenna remains stationary. The angular measurement system has an angle range from 0–45° on both sides, which means the sample had to be rotated once and two measurements were stitched together to obtain the scattering plots in both directions (±45°). The collimated beam has a diameter of 10 mm. The plotted intensity is the averaged intensity at this angle for all frequencies below the cut-off of the SPP-structure. All angular measurements are normalized to the 0° transmission of the same sample to avoid alignment dependent intensity differences. The frequency resolved angular plots are normalized with the bare antenna in the pivot point instead of the SPP waveguide, to adjust for eventual antenna characteristics.

## Supplementary information

Supplementary information
