# Supplementary material for: Scattering of spoof surface plasmon polaritons in defect-rich THz waveguides
Source: Sci Rep. 2019 Apr 18;9:6288. doi: 10.1038/s41598-019-42412-6 (PMC6472335; doi:10.1038/s41598-019-42412-6)
Supplement: Supplementary file 1 — Supplementary information [file 41598_2019_42412_MOESM1_ESM.pdf]

Scattering of spoof surface plasmon polaritons in defect-rich THz waveguides

Andreas K. Klein,[1],* Alastair Basden,[2] Jonathan Hammler, [1] Luke Tyas, [2] Michael Cooke, [1] , Claudio Balocco, [1] Dagou Zeze, [1] John M. Girkin,[2] Andrew Gallant[1]

Supplementary Material

Sample design and dispersion engineering

Our 3D printed SPP structures have a triangular cross-section as shown in the inset in fig. S1a. This is an unconventional shape as most 2D SPP structures have a rectangular cross-section (fig. S1b) with vertical sidewalls that are compatible with semiconductor manufacturing processes, but other shapes have been reported in the literature and can result in stronger confinement and longer propagation lengths [29-30]. The rectangular geometry also results in a simplification of the equations which describe the dispersion curve, since all edges of the structure are parallel to the electric field components. Nevertheless, a comparison to the well-explored rectangular design is worthwhile for understanding how the different geometric parameters influence the frequency behavior and the dispersion curve.

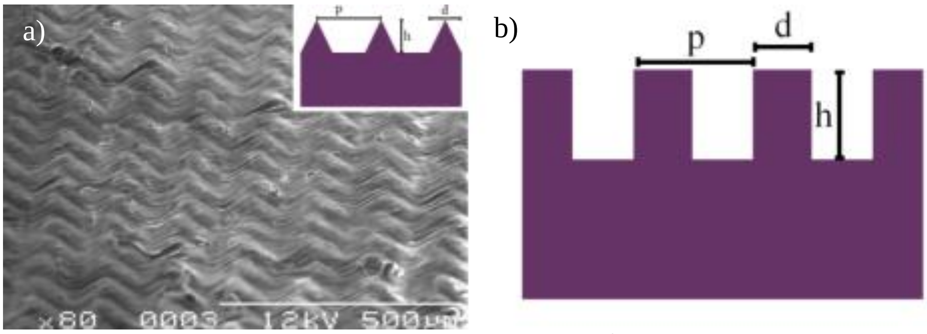

Fig. S1. The micrograph (a) shows the typical triangular cross-section of the 3D printed samples and the relevant geometrical parameters are indicated in the schematic in b. c) shows the crosscut of the rectangular structures with all relevant geometric parameters laying on the axes of a Cartesian coordinate system allowing a simpler description of the dispersion curve with equation 1.

Equation 1 (adapted from [31]) is an approximation of the dispersion curve which is found to be in good agreement with experimental results for structures with a rectangular cross section [32].

$$k_x = \frac{\omega}{c}\sqrt{\frac{(p-d)^2}{p^2}tan^2\left(\frac{\omega}{c}h\right)+1} \qquad (1)$$

The curvature of the dispersion relation is predominantly determined by the depth of the grooves, (h). Since the velocity of the SPP is proportional to the slope, this variable has the largest influence on the SPP propagation properties. The fraction of pitch (p) and groove thickness (d) allows one to fine tune the cut-off frequency, so a change in ratio results in a shifted curve with similar shape. To understand how these changes in geometry and the concomitant changes in the dispersion curves influence the transmission properties of the actual samples we compare the different dispersion curves (fig. S2a) with transmission spectra (fig S2a inset) obtained from Finite-difference time-domain (FDTD) simulations. Similar changes in dispersion curves and transmission spectra can be seen for a relative change of ~15% in pitch (p), which has only little influence other than on the cut-off frequency while the shape remains comparable, but an equal relative change in depth of the grooves (h) has a large influence on the slope with a smaller change in the cut-off frequency. The shapes of the transmission curves can be compared in a quantitative way using the usual metrics of the 3dB cut-off frequency and the roll-off, the linear part of the slope after the cut-off. When the pitch is changed the roll-offs change from -560 to -562 dB/THz with a shift in the 3 dB cut-off frequency of Δf≈0.08 THz (blue/green lines); but the slope changes significantly to -263 dB/THz with an equal change in height (blue/red lines). This indicates we can indeed use the transmission data to deduce the shape of the dispersion curve. Following on from this, we compare the effects of the same relative geometrical changes on the transmission properties of the triangular SPP structures (fig. 2b). The cut-off frequency of the triangular SPP structures is also most sensitive to the change in pitch (Δf≈0.1 THz, green/blue

lines), but in contrast to the rectangular structures, the slope also shows a significant change from -1000 to -1739 dB/THz. This indicates that not only the cut-off frequency changes with the pitch but also does the overall shape of the dispersion curve. On the other hand, the change in height results in a transmission curve shape which is comparable with the original curve, with a roll-off of -706 dB/THz and a smaller shift of Δf≈0.03 THz. This suggests that the design rules are not as straightforward for the triangular SPP structures as they are for the rectangular structures, where a change in a single parameter mostly changes only one characteristic of the transmission curve, but they do potentially add greater flexibility in design.

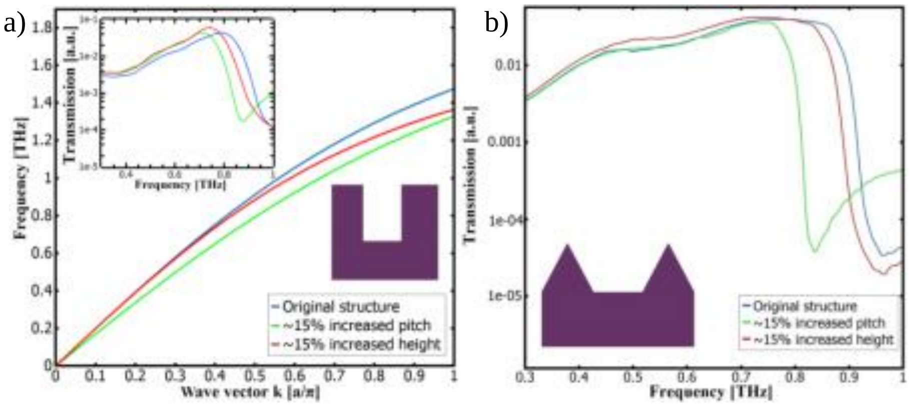

Fig. S2: The comparison of the dispersion curves of rectangular SPP structures (a, from eq. 1) with the transmission spectra (inset a, from FDTD) of the identical structures shows how the changes in the dispersion curve influence the transmission properties. With this, we can deduce the influence that the changes in the geometry of the triangular SPP structure have on the dispersion curve from its transmission spectra (b) by comparing the changes in transmission characteristic (cut-off frequency, roll-off). Original structures: p=150μm, d=50μm, and h=30μm, with p=175 μm for the increased pitch and h=35 μm for the increased height.

Power Spectral Density

The high defect density shown in the 3D visualization of the profilometer measurements of the original text is visible in the micrographs taken with a SEM in fig. S3 as well. For example, there are deformations to the surface along the ridges, probably originating from the stepwise printing process; defects where the grooves are damaged or incomplete; and debris, most likely originating from excess resin that forms irregularly shaped objects, on the surface. A chromatic lens profilometer has been used to provide a more quantitative analysis of the defect density. The surface profiles allow the average power spectral density (PSD) to be calculated which, in turn, gives access to the statistics of the surface roughness.

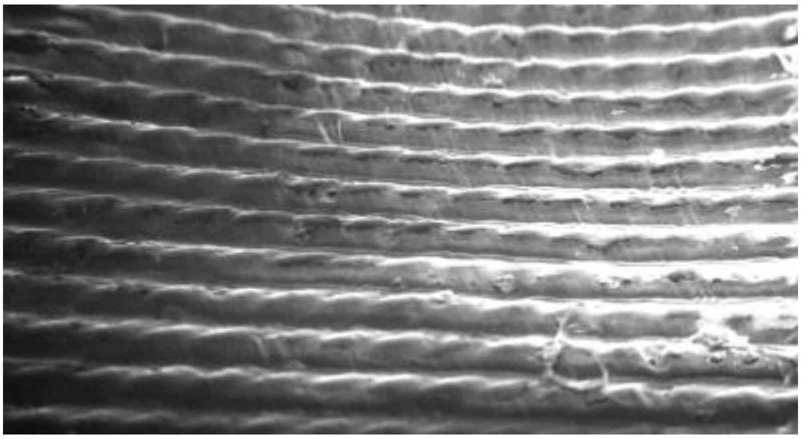

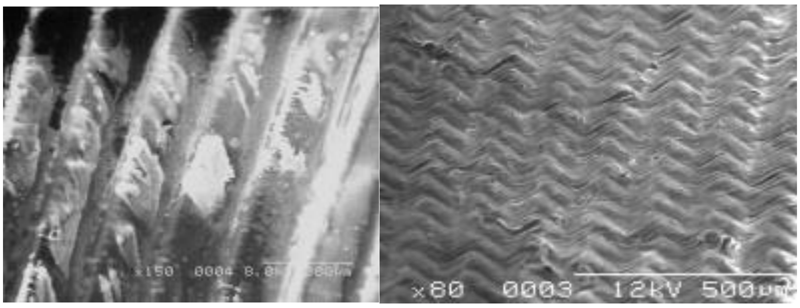

Fig S3: Micrographs from the sample surfaces. Top: The ridges of the structures show a corrugation along the direction of printing, probably caused by the stepping of the printing process. Bottom left: Low surface quality like the incomplete printed groves are an indicator that the feature sizes push the limits of the technique used. Bottom right: Debris formed by excess resin.

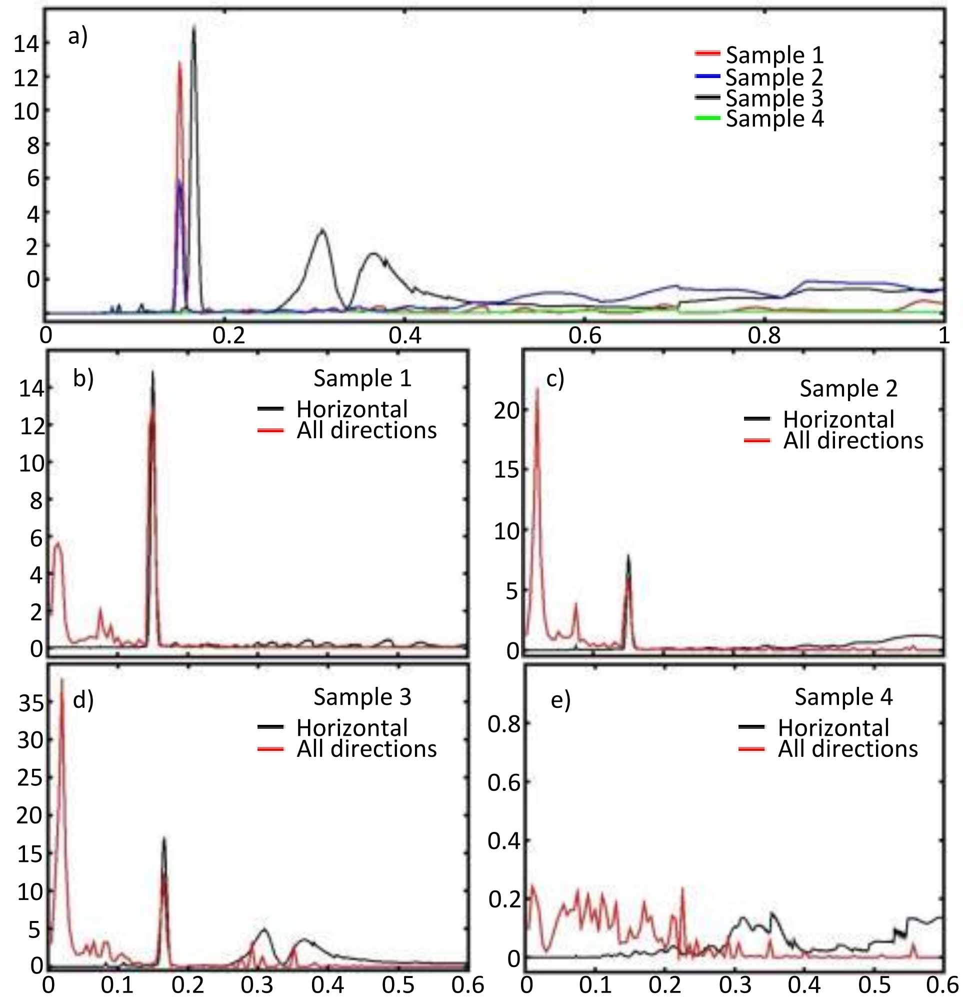

Fig S4: a) Averaged Power Spectral Density (PSD) including only horizontal wave vectors that go across the corrugation of the SPP structure. The first three samples are in good agreement with the target values listed in table 1; the fourth sample does not have any prominent features. b-e) PSD only including the horizontal wave vectors in comparison with the PSD including wave vectors of all directions. While the periodicity of the structure dominates along the horizontal axis, the high amount of roughness and defects becomes apparent when considering all directions.

In fig. S4a we can see that the periodicities of sample 1-3 are in good agreement with the target parameters listed in table 1, with 150 µm for sample 1 and 2 and 166 µm for sample 3. Additionally, we can see two other broad peaks for structure 3 at roughly double the wavelength of the desired periodicity. The 3D visualization of the surface in fig. 2a (in the main text) indicates that this might be caused by the variation in the height of every second groove. Furthermore, fig. S4 also shows that all structures have an above zero amplitude at most other wavelengths, indicating a broad range of defects. This is affirmed when comparing the horizontal component PSD

with the PSD that includes all directions for each structure (Fig. S4 b-e). Here we see that besides the prominent peak at the wavelength of the periodicity, there is a very well defined feature at shorter wavelengths and some small peaks scattered over the spectrum. The 3D maps and SEM micrographs show that this part of the PSD is indeed caused by the roughness or defects on the surface. The larger peak at the shortest wavelengths is most likely due to the general surface quality whereas the other smaller peaks in the spectrum are caused by other defects, e.g. debris or incomplete structures as seen in the micrographs in fig. S3.

Knife-edge scattering

A propagating SPP is described by

$$E(x) = E_0 e^{ik_x x} = E_0 e^{ik'_x x} e^{-k''_x x}, \qquad (2)$$

Where k is the wave vector and x denotes the direction of propagation. The propagation length is defined as

$$L_x = \frac{1}{2k''_x}, \qquad (3)$$

While the extension of the electric field of the SPP in the direction z, perpendicular to the sample surface, is described as an evanescent wave by

$$E(z) = E_0 e^{-k''_z z}. \qquad (4)$$

And the characteristic length of the extension of the electric field, the confinement, is defined as

$$L_z = \frac{1}{|k''_z|}. \qquad (5)$$

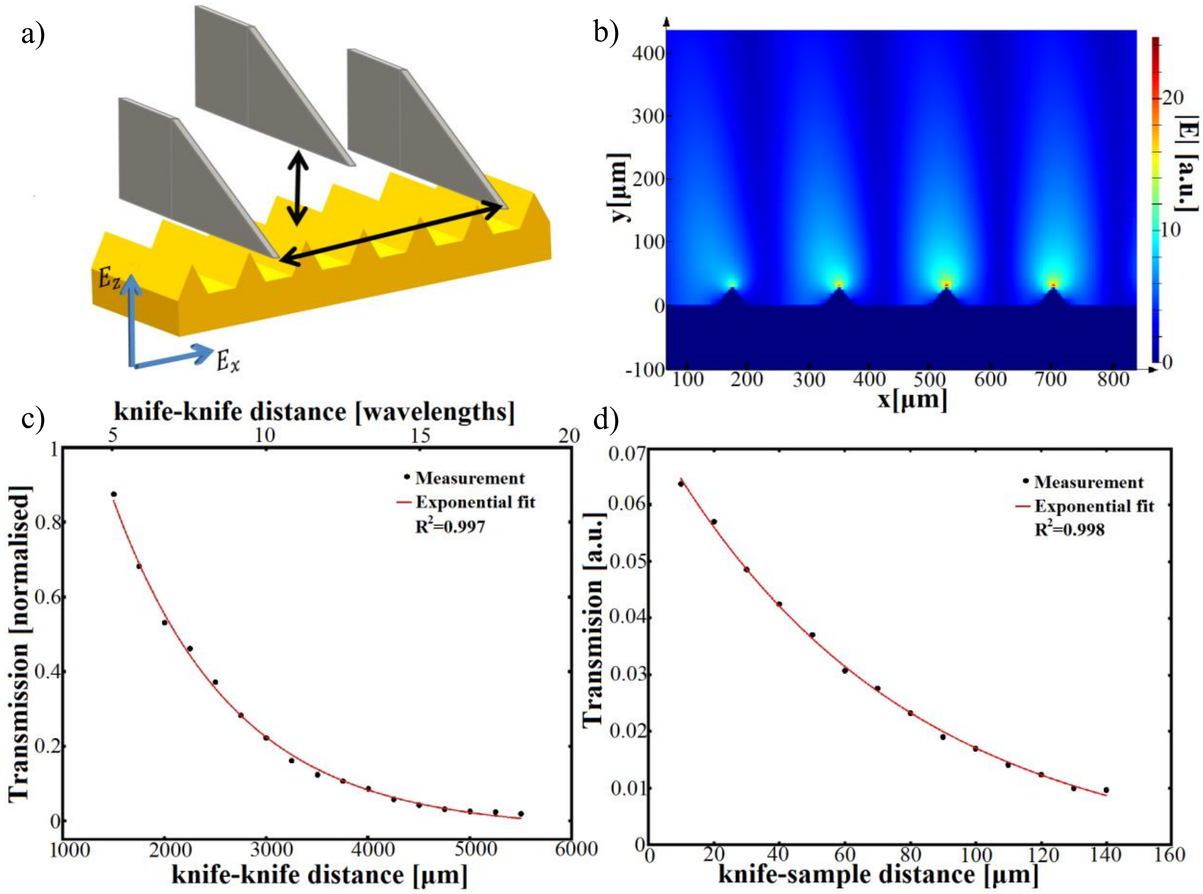

Fig. S5. a) Schematic of the knife-edge scattering experiments. The variable knife-knife distance for the propagation length along the x-axis and the variable knife-sample distance along the z-axis are indicated by arrows. b) Electric field distribution over the sample surface from FDTD simulations. The experimental data (dots) to measure the c) propagation length and d) confinement and the respective exponential fits (red line). Table 1 shows results of the fits including associated errors.

The intensity at frequencies near to the cut-off frequency is plotted over the respective length change, and a subsequent fit leads to the characteristic lengths. As seen in fig. S5, the experimental data fit the exponential decay well (all R^2>0.995). The results of the fittings can be found in table 1.

References

[29] X. Liu, L. Zhu, Q. Wu and Y. Feng, "Highly-confined and low-loss spoof surface plasmon polaritons structure with periodic loading of trapezoidal grooves," AIP Adv. 5 (2015).

[30] X. Gao, L. Zhou and T. Jun Cui, "Odd-Mode Surface Plasmon Polaritons Supported by Complementary Plasmonic Metamaterial," Sci. Rep.5 (2015).

[31] Q. Gan, Z. Fu, Y. J. Ding and Filbert J. Bartoli, "Ultrawide-Bandwidth Slow-Light System Based on THz Plasmonic Graded Metallic Grating Structures," Phys, Rev. Lett. 100, 256803 (2008).

[32] A.K. Klein, Y. Pan, C. Balocco, D. Zeze, A. J. Gallant, "Micro fabricated spoof surface plasmon polariton structures for THz applications," IRMMW-THz (IEEE, 2015).
